# Draft genome sequence of endolichenic fungus *Dothichiza* sp. strain YAFEF048, isolated from *Usnea longissima* on Haba Snow Mountain

**DOI:** 10.1128/MRA.01011-23

**Published:** 2023-11-16

**Authors:** Jiansheng Wei, Xiaolong Yuan, Yunfen Geng, Wei Bi, Yi Wang

**Affiliations:** 1 Haba Snow Mountain Provincial Nature Reserve Management and Protection Bureau, Diqing, China; 2 Laboratory of Forest Plant Cultivation and Utilization, Yunnan Academy of Forestry & Grassland, Kunming Yunnan, China; University of California, Riverside, Riverside, California, USA

**Keywords:** *Dothichiza* sp, genome, *Usnea longissima*

## Abstract

In this study, we report a draft genome sequence of the endolichenic fungus *Dothichiza* sp. strain YAFEF048, isolated from the lichen *Usnea longissim*a on Haba Snow Mountain. The genome resource will support future research into the endolichenic lifestyle and potential secondary metabolite diversity of this fungus.

## ANNOUNCEMENT

Lichens are a type of mutualistic symbiosis between obligate fungi and either algae or cyanobacteria (1). Lichens are typically rich in endolichenic fungi (2, 3). Endolichenic fungi often display antibacterial, antifungal, antiviral, and antimalarial activity and are a new and rich source of bioactive secondary metabolites (4, 5).

The endolichenic fungus *Dothichiza* sp. (Ascomycota; Dothidiomycetes) strain YAFEF048 was isolated from the lichen *Usnea longissima*. The isolation process was as follows. *U. longissimi* was collected from the bark of *Pinus densata* on Haba Snow Mountain, Yunnan Province, China (27°30'24" N, 100°08'44" E), in 2020. The thallus was washed by soaking it in tap water for 24 hours and then rinsing it with sterile water. A tissue grinder was used to grind the thallus, after which sterile water was added to obtain a slurry. Finally, the thallus slurry was further diluted and spread onto a solid malt-yeast medium (Difco, Lawrence, KS, USA) containing 100 µg/mL of ampicillin, kanamycin, and streptomycin, respectively. After 3 days of incubation (28°C), single colonies were transferred to a fresh medium. One single colony (strain YAFEF048) was identified by sequencing the internally transcribed spacer (ITS) region as belonging to the genus *Dothichiza* (6), and the ITS sequence was deposited in GenBank (accession number: OR447492). *Dothichiza* sp. strain YAFEF048 was deposited in the China Center for Type Culture Collection (deposit number: CCTCC M 20211345).

The mycelia of *Dothichiza* sp. strain YAFEF048 were harvested for DNA extraction after cultivation at 28°C for 5 days on a rotary shaker at 150 rpm. Genomic DNA was extracted using a DNeasy minikit (Qiagen, Valencia, CA, USA). The library preparation was performed using the TruSeq Nano DNA LT Library Preparation Kit (New England Biolabs, USA), and insert sizes of 400 bp were retained. The library was sequenced on the Illumina NovaSeq platform with a 2 × 150 bp paired-end configuration. A total of 28,069,536 raw reads were obtained (Table 1). The high-quality cleaned sequences (3.81 Gb; coverage, 122×) were obtained by trimming adapter sequences using AdapterRemoval version 2 (7). Default parameters were used for all software unless otherwise specified. The genome was assembled using SPAdes version 3.10.1 (8). The genome size of strain YAFEF048 was 31,278,652 bp with 79 scaffolds. The length of the longest scaffold was 2,423,348 bp, with an N50 value of 1,156,424 bp and an N90 value of 340,043 bp. The GC content was 52.589%. The gene completeness of the genome assembly was assessed using BUSCO version 5.4.2 (9), and the completeness value was 99.5%.

**TABLE 1:** Genome sequencing statistics

Parameter	Value
Raw reads	
Reads number	28,069,536
Coverage	122×
Genome assembly	
Total base	31,278,652
Total scaffolds	79
Max sequence length	2,423,348 bp
N50	1,156,424 bp
N90	340,043 bp
Genome assembly assessment	
Complete BUSCOs	99.50%
Complete and single-copy BUSCOs	99.40%
Complete and duplicated BUSCOs	0.10%

## Data Availability

This Whole Genome Shotgun project has been deposited at DDBJ/ENA/GenBank under the accession number JAVISA000000000, BioProject accession number PRJNA1005758, BioSample accession number SAMN36998855, and SRA accession number SRX21354215.
